# BERMAD: batch effect removal for single-cell RNA-seq data using a multi-layer adaptation autoencoder with dual-channel framework

**DOI:** 10.1093/bioinformatics/btae127

**Published:** 2024-03-04

**Authors:** Xiangxin Zhan, Yanbin Yin, Han Zhang

**Affiliations:** Department of Intelligence Engineering, College of Artificial Intelligence, Nankai University, Tianjin 300350, China; Department of Food Science and Technology, University of Nebraska – Lincoln, Lincoln, NE 68588, United States; Department of Intelligence Engineering, College of Artificial Intelligence, Nankai University, Tianjin 300350, China

## Abstract

**Motivation:**

Removal of batch effect between multiple datasets from different experimental platforms has become an urgent problem, since single-cell RNA sequencing (scRNA-seq) techniques developed rapidly. Although there have been some methods for this problem, most of them still face the challenge of under-correction or over-correction. Specifically, handling batch effect in highly nonlinear scRNA-seq data requires a more powerful model to address under-correction. In the meantime, some previous methods focus too much on removing difference between batches, which may disturb the biological signal heterogeneity of datasets generated from different experiments, thereby leading to over-correction.

**Results:**

In this article, we propose a novel multi-layer adaptation autoencoder with dual-channel framework to address the under-correction and over-correction problems in batch effect removal, which is called BERMAD and can achieve better results of scRNA-seq data integration and joint analysis. First, we design a multi-layer adaptation architecture to model distribution difference between batches from different feature granularities. The distribution matching on various layers of autoencoder with different feature dimensions can result in more accurate batch correction outcome. Second, we propose a dual-channel framework, where the deep autoencoder processing each single dataset is independently trained. Hence, the heterogeneous information that is not shared between different batches can be retained more completely, which can alleviate over-correction. Comprehensive experiments on multiple scRNA-seq datasets demonstrate the effectiveness and superiority of our method over the state-of-the-art methods.

**Availability and implementation:**

The code implemented in Python and the data used for experiments have been released on GitHub (https://github.com/zhanglabNKU/BERMAD) and Zenodo (https://zenodo.org/records/10695073) with detailed instructions.

## 1 Introduction

Single-cell RNA sequencing technology combined with computational biological analysis can reveal the specific and independent transcriptome information of cells in heterogeneous states (Kharchenko 2021). However, with the growth of more and more scRNA-seq techniques, the scale of generated datasets is also increasing. Large-scale scRNA-seq datasets generated from different experiment platforms usually contain batch-specific systematic variations, the so-called ‘batch effect’ (Tran *et al.* 2020). In order to accurately carry out various related cell and gene research on datasets described above, it is very crucial to first conduct batch effect removal.

There are already research efforts on batch effect removal. Linear model is the first method proposed to remove batch effect from data. In fact, it was originally used to process microarray gene expression data. Smyth and Speed (2003) proposed limma; it fits the input data into a linear model to capture the batch effect, which will be subtracted later from the original data to obtain the batch corrected gene expression matrix. Combat (Smyth and Speed 2003) is another method proposed for microarray gene expression data, and Risso *et al.* (2018) applied it to scRNA-seq data. Combat uses an empirical Bayes method to fit the standardized input data to standard distributions for estimating the batch effect, and then utilizes the obtained batch correction operator to conduct batch effect removal. With continuous growth of the scale of scRNA-seq datasets, linear models are difficult to handle such complex and highly nonlinear data because of its limited representation ability.

Haghverdi *et al.* (2018) first proposed to use MNNs between batches to remove batch effect. They use the identified MNN pairs to compute the batch correction vector and apply it to all cells in the dataset. Lately, Lun (2019) improved the above method and proposed fastMNN. The promotion is that MNN pairs are identified in dimension-reduced PCA space, thus avoiding the high computational cost brought by the original high-dimensional gene expression space. BBKNN (Polański *et al.* 2020) uses the neighborhood graph of cells in batches to perform data integration and batch correction. Scanorama (Hie *et al.* 2019) is analogous to computer vision algorithms for panorama stitching, where it automatically identifies scRNA-seq datasets containing cells with similar transcriptional profiles and leverages those matches for batch correction. All MNN-based methods rely on the accuracy of MNN pairs’ recognition and tend to work correctly only when the cell type compositions across batches are relatively similar.

There are some approaches based on component analysis and dimension reduction, Seurat 3 (Stuart *et al.* 2019) is a typical one of them. It uses canonical correlation analysis (CCA) for dimension reduction, and identifies MNN pairs in the obtained dimension-reduced CCA space to calculate the correction vector. Another method of this approach is Harmony (Korsunsky *et al.* 2019), which first projects all batches into a dimension-reduced space with the help of PCA, and then iterates between cell clustering and batch correction until convergence. Poličar *et al.* (2023) first constructs a t-SNE embedding of one dataset as reference, then embeds another dataset to the reference-defined low-dimensional space to remove batch effect. However, these methods are highly dependent on the quality of the dimension reduction work and thus only work well under specific circumstances.

In recent years, there have been some deep learning-based methods dedicated to removing batch effect. scVI (Lopez *et al.* 2018) utilizes a deep generative model to approximate the underlying distribution of input gene expression values, and the output is subsequently used to deal with batch effects. BERMUDA (Wang *et al.* 2019) aligns similar clusters in a low-dimensional space by training a deep autoencoder to achieve batch effect removal. HDMC (Wang *et al.* 2022) address batch effect problem through a hierarchical distribution-matching framework as well as contrastive learning. iMAP (Wang *et al.* 2021) removes batch effect based on both deep autoencoders and generative adversarial networks. mtSC (Duan *et al.* 2021) regards each dataset as an individual task to train a multitask-based deep neural network for single-cell alignment. Among these methods, scVI and BERMUDA either pay excessive attention to bridge the distribution difference between datasets or focus too much on the alignment of similar cell clusters, which may lead to the loss of independence and specificity of individual dataset. On the other hand, although HDMC uses contrastive learning to address over-correction, it essentially processes different batches jointly without considering batch specificity. iMAP needs to obtain MNN pairs between batches before batch correction step, so it still relies on the accuracy of MNN pairs’ recognition. As for mtSC, it is proposed for dataset assignment instead of batch correction or data integration.

As discussed above, some previous methods rely on assumptions that do not always meet or cannot deal with highly nonlinear data, which may lead to under-correction. However, other methods pay too much attention to reduce the difference between batches and ignore some important exclusive information of an individual dataset, which may cause over-correction. To address these concerns, we propose a novel multi-layer adaptation autoencoder with dual-channel framework, attempting to develop a unified framework to simultaneously tackle the under-correction and over-correction problems in batch effect removal.

For our BERMAD model, in order to integrate datasets from different experiment platforms more completely and accurately, we design a multi-layer adaptation architecture, which carries on distribution matching of different dimensions in different feature layers of the autoencoder, and then generates a batch effect corrected embedding for downstream tasks. At the same time, for explicitly considering the specificity and independence of an individual dataset, we propose a dual-channel framework. In detail, we train network parameters for different batches separately, so that the autoencoders processing different batches have the same model architecture but have unshared weights, thus solving the over-correction problem caused by the loss of dataset specificity. Experiments on simulated dataset and real scRNA-seq datasets have proved our method’s effectiveness and superiority over other state-of-the-art methods.

Our method has two advantages compared with previous methods: (i) the novel multi-layer adaptation architecture can reduce the distribution difference of similar cell clusters between different batches more effectively. The outputs on different layers of a deep autoencoder have different feature dimensions and contain information of different granularity, allowing for comprehensive distribution matching at different levels, thereby producing to more accurate batch correction results. (ii) The dual-channel framework of BERMAD can preserve the unique biological signals of a single dataset, such as unshared cell types. The same network architecture but independently trained parameters ensure correct clustering and integration, thus effectively reducing over-correction.

## 2 Materials and methods

**Figure 1.**
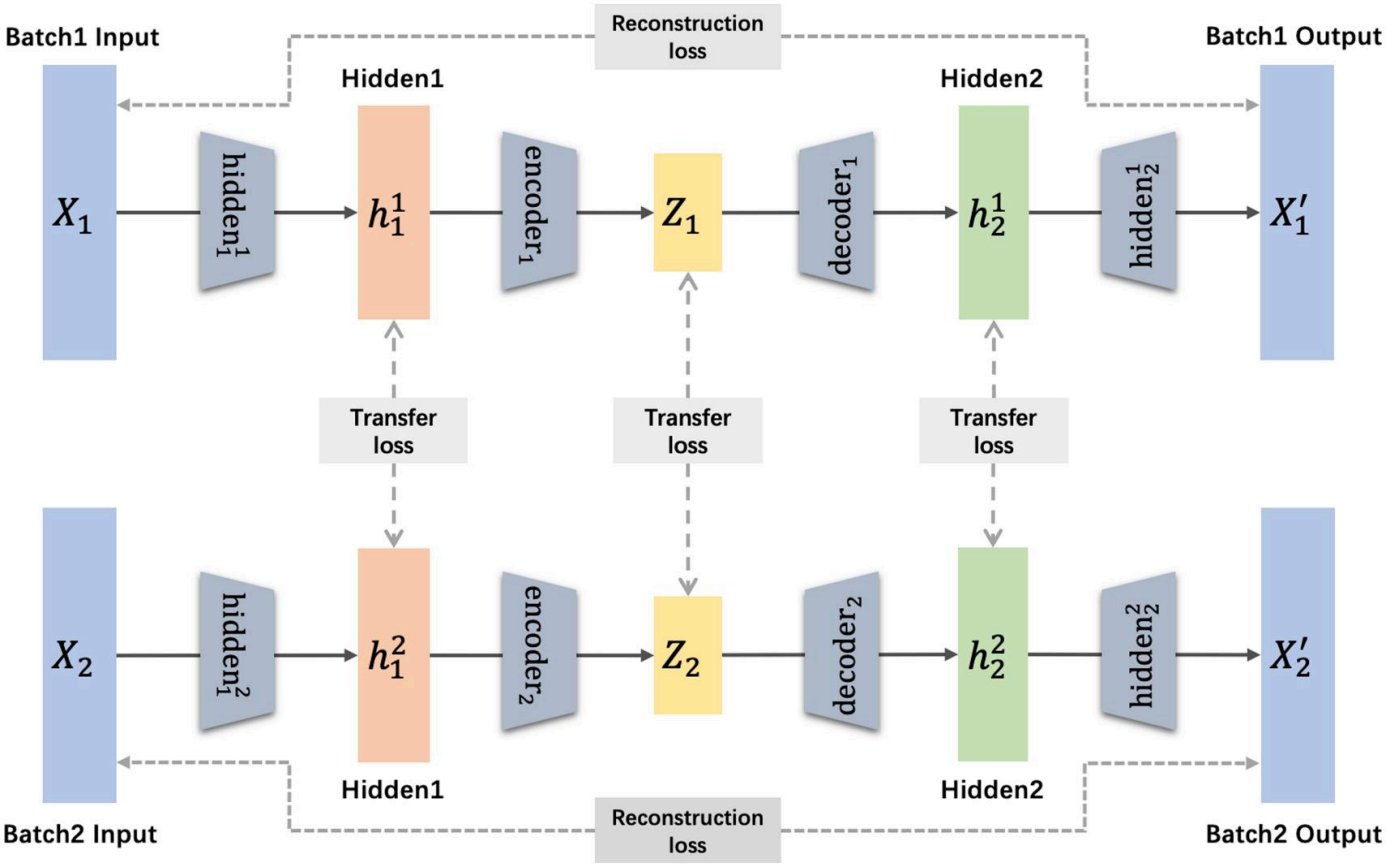
Overall framework of BERMAD. The basic architecture is a deep autoencoder with four components and three intermediate outputs. The inherent reconstruction loss of the autoencoder is used to obtain the appropriate low-dimensional representation of the input data, and the transfer loss of three intermediate outputs is used to match the distribution between two batches. Besides, the graph representation that two batches have the same but separate architecture reflects our dual-channel framework.

The overall architecture of our method is shown in Fig. 1. Let $X_{1}=\{x_{1},\ldots ,x_{m}\}$ and $X_{2}=\{x_{1},\ldots ,x_{n}\}$, respectively, represent the input of scRNA-seq dataset from two batches, $X_{1}^{\prime }=\{x_{1}^{\prime },\ldots ,x_{m}^{\prime }\}$ and $X_{2}^{\prime }=\{x_{2}^{\prime },\ldots ,x_{n}^{\prime }\}$ are their reconstructions. The goal is to train a deep autoencoder to obtain the batch corrected low-dimensional embeddings $Z_{1}$ and $Z_{2}$ of the original data, which will be combined into the final output *Z* after the task is completed. Specifically, we use the inherent reconstruction loss of the autoencoder to obtain the appropriate low-dimensional embedding of the original high-dimensional gene expression data. Then we calculate the transfer loss on the output of three hidden layers $h^{1}$, $h^{2}$ and *z* with different dimensions simultaneously to reduce the distribution difference between two batches. Detailed description about the workflow and the diagram of our method can be found in Supplementary material SI.

In fact, our design was inspired by the recent advance in the research of domain adaptation, which can be successfully transferred to batch effect removal tasks we are interested in. Domain adaptation is a subfield within machine learning that attempts to generalize the model learned from the source domain to the target domain. It allows the model transfer and generalization by minimizing the distribution difference between domains (Farahani *et al.* 2021). The mainstream method of domain adaptation is to perform distribution matching between source domain and target domain, similar to that we perform distribution matching between different batches. To our knowledge, we are the first to apply multi-layer adaptation to batch correction and design a corresponding approach for batch effect removal.

### 2.1 Basic model

We choose deep autoencoder as the basic model for our method, since it is an unsupervised learning method widely used in domain adaptation research and has excellent performance in data dimension reduction, feature extraction and data reconstruction (Zhang *et al.* 2020). Specifically, the basic model of our framework is a deep autoencoder consisting of an input layer, three hidden layers with dimensions of 200, 20, and 200, and an output layer, which is a common setting in domain adaptation. The reconstruction loss between the original input and the reconstruction output is calculated to obtain the appropriate low-dimensional embedding of the original gene expression data, so the first term of the loss function of our method is the inherent reconstruction loss of the autoencoder:

(1) $$\begin{array}{c} L_{\mathrm{rec}}=\sum_{i=1}^{n}{\left\|x_{i}-x_{i}^{\prime }\right\|}_{2}^{2}, \end{array}$$

where $x_{i}$ is the original input sample and $x_{i}^{\prime }$ is its reconstruction.

### 2.2 Multi-layer adaptation

In order to reduce the distribution difference between clusters of the same type from different batches for accurate batch effect removal, we conduct distribution matching on the intermediate outputs of the deep autoencoder. We choose the maximum mean discrepancy (MMD) (Gretton *et al.* 2012) between different batches as the measure of distribution difference, which is a common choice in domain adaptation. MMD is a nonparametric method to measure the distance between embeddings of the probability distributions in a reproducing kernel Hilbert space (RKHS), and performs well in many deep transfer learning tasks (Ghifary *et al.* 2014, Long *et al.* 2017, Ying *et al.* 2018). The detailed introduction of MMD is shown in Supplementary materials SII.

#### 2.2.1 The calculation of transfer loss

In order to correctly calculate the MMD between cell clusters of different batches before distribution matching, we need to first divide each batch into several clusters individually. To this end, we cluster the datasets of each batch separately using the Seurat package provided by Butler *et al.* (2018), which perform graph-based clustering on the data with Louvain algorithm. Since the cell types contained in different batches may not be identical and only the distribution difference between similar cell clusters contribute to the task, we need to filter out similar pairs of cell clusters between batches. Therefore, we run the MetaNeighbor algorithm of Crow *et al.* (2018) to calculate the similarity score of the cell cluster pairs between different batches. We briefly describe the working principle of MetaNeighbor in Supplementary material SIII. Only the cell cluster pairs with the similarity score larger than the similarity threshold (0.85 as the default) are included in the distribution discrepancy calculation. In general, our MMD-based transfer loss is computed as follows:

(2) $$\begin{array}{c} \mathrm{transfer}\_\mathrm{loss}=\sum_{c_{i}\in X_{1},c_{j}\in X_{2}}s_{i,j}\cdot \mathrm{MMD}(e(c_{i}),e(c_{j})), \end{array}$$

where $c_{i}$ and $c_{j}$ are the cell clusters of batch 1 and batch 2 respectively, $e(c_{i})$ and $e(c_{j})$ are their intermediate output embeddings, and s(i,j) is the similarity score between $c_{i}$ and $c_{j}$. We binarize it according to our requirement:

(3) $$\begin{array}{c} s_{i,j}=\{\begin{array}{cc} 1, & s_{i,j}\geq \mathrm{thr}\\ 0, & \text{otherwise}, \end{array} \end{array}$$

where *thr* is the similarity threshold used to filter out similar cell cluster pairs, set to 0.85 by default.

#### 2.2.2 Design of multi-layer adaptation

Since a deep autoencoder has multiple intermediate layers from input to output, it’s a new problem to determine which layers’ distribution matching should be performed, i.e. which layers’ transfer loss should be included in the loss function of our framework. Yosinski *et al.* (2014) pointed out that the feature transferability of different layers in deep neural network will vary greatly with domain discrepancy, so the performance of single-layer adaptation is often limited. In order to solve this problem, Long *et al.* (2015) proposed deep adaptive network (DAN). DAN can perform distribution matching between source domain and target domain, where it learns transferable features with statistical guarantees by calculating transfer loss on multiple intermediate layers of the deep neural network.

#### 2.2.3 Transfer loss of BERMAD

Domain adaptation reduces domain discrepancy by performing distribution matching between domains, and often chooses MMD to measure distribution difference. If source domain and target domain contain datasets of similar types or tasks, we only need to mitigate domain shift to achieve generalization. Similarly, we also perform distribution matching between batches with MMD to remove batch effect. Besides, scRNA-seq data from different batches are measured for the same biological problem, which can be integrated for downstream analysis only after removing batch effect. Inspired by above discussion, we design the multi-layer adaptation architecture of BERMAD. Specifically, we calculate transfer loss on the embeddings $h^{1}$, $h^{2}$, and *z* of the three intermediate layers in the deep autoencoder as shown in Fig. 1 according to Equation (2). Then we perform distribution matching on embeddings of different dimensions, to more completely reduce the distribution differences of similar cell clusters between batches. Finally, the second term of our framework’s loss function is a weighted sum of three calculation results of intermediate transfer loss:

(4) $$\begin{array}{c} L_{\mathrm{tran}}=\alpha \cdot L_{h^{1}}+\beta \cdot L_{h^{2}}+\gamma \cdot L_{z}, \end{array}$$

where $L_{h^{1}}$, $L_{h^{2}}$ and $L_{z}$ represent the single-layer transfer loss on the intermediate layer $h^{1}$, $h^{2}$ and *z*, respectively. α, β and γ are hyperparameters for balancing the transfer loss items of different layers, where they may have different optimal values on different datasets but generally robust. To verify the robustness, we conduct comparative experiments with different hyperparameter values on the simulated dataset and the real dataset Pancreas, and present the results in Supplementary material SIV. At the same time, considering the convergence of the algorithm, their values should not be too large, which are set to 0.2, 0.2, 0.2 by default.

#### 2.2.4 Total loss of BERMAD

The total loss function of our framework is formed by combining the reconstruction loss inherent in deep autoencoder and the multi-layer adaptation transfer loss designed by us:

(5) $$\begin{array}{c} L=L_{\mathrm{rec}}+\lambda \cdot L_{\mathrm{tran}}, \end{array}$$

$L_{\mathrm{rec}}$ is the reconstruction loss used to obtain the appropriate low-dimensional representation of the original data. $L_{\mathrm{tran}}$ is the transfer loss used to match the distribution of similar cell clusters between batches, and λ is a hyperparameter balancing those two components. We follow the strategy proposed by Ganin and Lempitsky (2015) to gradually increase λ from 0 to 1:

(6) $$\begin{array}{c} \lambda =\frac{2}{1+e^{-\frac{10p}{n}}}-1, \end{array}$$

where *n* is the number of total epochs and *p* is the current epoch. With this parameter setting, we can make the network focuses on finding the appropriate low-dimensional representation of data in the early training process, and focus on matching similar cell clusters in the later training process.

### 2.3 Dual-channel framework

There is another common and thorny problem in the field of batch correction, which is over-correction caused by too much attention to the distribution matching between batches. For example, although most of the cell types contained in scRNA-seq data from different batches are the same or similar, there are still some unshared cell types present within an individual dataset in some real tasks. If we ignore this kind of specificity of datasets, it may lead to incorrect alignment of dissimilar cell clusters after integration. Therefore, we should avoid such over-correction.

This phenomenon also exists in domain adaptation. In some cases, the domain invariance caused by over-mitigating domain differences might be detrimental to discriminative power of the method, leading to declined performance (Rozantsev *et al.* 2018). Rozantsev *et al.* (2018) proposed a two-stream architecture that explicitly models the domain shift, where each stream serves for a single domain (source domain or target domain). This allows the models able to handle data from different domains with the same network architecture but different parameters. At the same time, a specifically designed loss is used to constrain the differences between domains, so that the parameters of the models will not be too different from each other.

Inspired by the above method, we propose a dual-channel framework to address the problem of over-correction and the loss of dataset specificity for batch correction. We make the models handle datasets from different batches independently of each other (as depicted by Fig. 1). In other words, the deep autoencoders encoding different datasets have the same architecture but different parameter values. This structural separation helps us explicitly model the difference between batches and the specificity of datasets. Since a single deep autoencoder is trained independently, its reconstruction loss is also optimized independently, which helps the low-dimensional embedding of data more completely retain the unshared information. At the same time, the transfer loss between data embeddings in different channels also plays an important role in constraining the parameters between batches not to differ too much, preventing the failure of data integration. Through such dual-channel framework, we can accurately align similar cell clusters between datasets while maintaining the independence of unshared type cell clusters.

## 3 Results

### 3.1 Simulated data

First, we evaluated our method on a simulated dataset generated using the splatter package in R (Zappia *et al.* 2017). The dataset consists of two batches (denoted as Batch 1 and Batch 2); Batch 1 contains 2000 cells, and Batch 2 contains 1000 cells. Since linear methods developed in early time were not designed for highly nonlinear scRNA-seq data, so we exclude them from method comparison. We compared our method with MNN (Haghverdi *et al.* 2018), BBKNN (Polański *et al.* 2020), Scanorama (Hie *et al.* 2019), Harmony (Korsunsky *et al.* 2019), scVI (Lopez *et al.* 2018), BERMUDA (Wang *et al.* 2019), HDMC (Wang *et al.* 2022), and iMAP (Wang *et al.* 2021), which are representative methods in their respective categories and have been widely used in recent years. We present the experiment results qualitatively and quantitatively for more comprehensive comparison.

**Figure 2.**
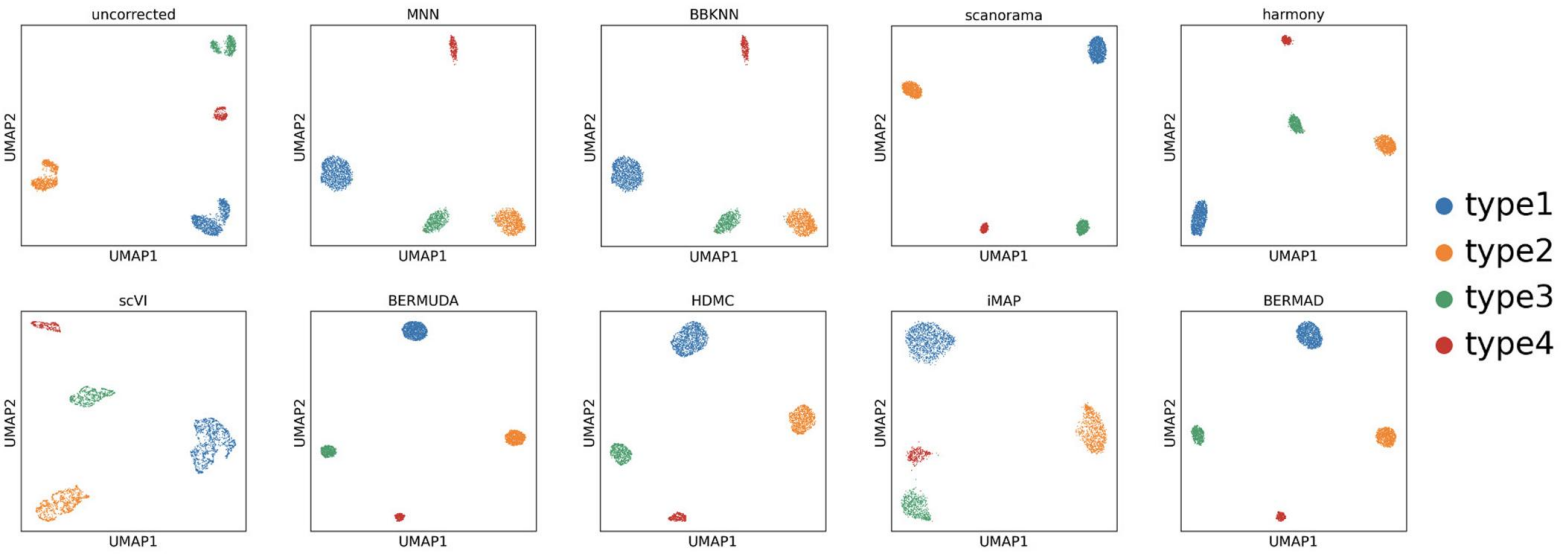
Visualization for batch effect removal on simulated dataset.

The qualitative visualization results on the simulated dataset are shown in Fig. 2. It shows a significant margin between cell clusters of the same type from different batches, which is known as batch effect. Not removing batch effect will lead to lower accuracy of subsequent downstream analysis such as cell clustering. It can be seen that all the methods we selected can correctly perform data integration and batch correction on the simulated dataset, but produce clusters with different densities. Among all methods, BERMAD and BERMUDA have the most impressive visualization results as they generate compact and independent cell cluster structures. In this article, we only illustrate qualitative figures dyed by cell type. All the qualitative visualization results dyed by batch ID are presented in Supplementary material SV.

For quantitative evaluation, we choose divergence, silhouette and Adjusted Rand Index (ARI) to evaluate our method and other comparison methods from different perspectives. Divergence is used to measure the distribution discrepancy between batches, so a lower value is better. Silhouette evaluates whether dissimilar cell clusters still maintain good separation after integration, so a higher value is preferred. A higher ARI means that the clustering result is closer to ground truth, which also means better batch correction performance. The detailed explanations and calculation formulas of these three metrics are shown in Supplementary material SVI. The quantitative calculation results of three performance evaluation metrics are presented in Table 1. Due to the simple composition of the simulated dataset, the ARI of all methods is roughly the same, so we only analyze the divergence and silhouette. From the table, it can be seen that BERMAD has the lowest divergence and the highest silhouette, showing the effectiveness and superiority of our method. Note that most methods cannot consider both divergence and silhouette simultaneously, as they are conflicting performance evaluation metrics, while BERMAD can achieve optimal results on both metrics simultaneously. Meanwhile, although BERMUDA has comparative visualization result to BERMAD, its metric calculation results are not as good as ours. On this dataset, we set thr=0.90, α=β=γ=0.3.

**Table 1.** Calculation results of evaluation metrics on simulated dataset.

Method	Divergence	Silhouette	ARI
Uncorrected	3.96±0.06	0.34±0.00	1.00±0.00
MNN	0.52±0.01	0.33±0.00	0.99±0.00
BBKNN	0.52±0.01	0.33±0.00	0.99±0.00
Scanorama	0.02±0.00	0.43±0.00	1.00±0.00
Harmony	0.10±0.03	0.34±0.00	1.00±0.00
scVI	0.42±0.06	0.77±0.03	0.99±0.01
BERMUDA	0.02±0.01	0.83±0.01	1.00±0.00
HDMC	0.04±0.01	0.90±0.00	1.00±0.00
iMAP	0.46±0.07	0.45±0.02	0.98±0.02
BERMAD	**0.01 ± 0.00**	**0.95 ± 0.00**	1.00±0.00

The optimal results are highlighted in bold font.

### 3.2 Pancreas data

To compare our method with others on real data, we further applied BERMAD to human Pancreas scRNA-seq dataset. The dataset consists of two batches called Muraro (Muraro *et al.* 2016) and Baron (Baron *et al.* 2016), generated by different sequencing techniques CEL-Seq2 and droplet RNA seq, and each batch contains eight cell types. In order to compare the performance of different methods more comprehensively, we divided the experiment on this dataset into three parts. In the first part, we performed batch correction on the whole dataset, which corresponds to the situation that batches have identical cell type composition. We refer to it as Pancreas_Same, where batch Muraro contains 2042 cells, and batch Baron contains 8012 cells. In the second part, we removed the alpha and beta types from Baron, which corresponds to the situation where there are unshared cell types between batches. We refer to it as Pancreas_Diff, where batch Muraro still contains 2042 cells, while batch Baron contains 3161 cells. In the third part, we introduced another dataset Seger (Segerstolpe *et al.* 2016) with a different sequencing technique called SMART-Seq2. We removed the alpha type from Muraro and removed the beta type from Seger, which is also for unshared cell types between batches. We refer to it as Pancreas_Diff2, where batch Muraro contains 1230 cells and batch Seger contains 1791 cells. The experimental results on Pancreas_Same are as follows, and the experimental results on other two datasets are shown in Supplementary material SVII.

**Figure 3.**
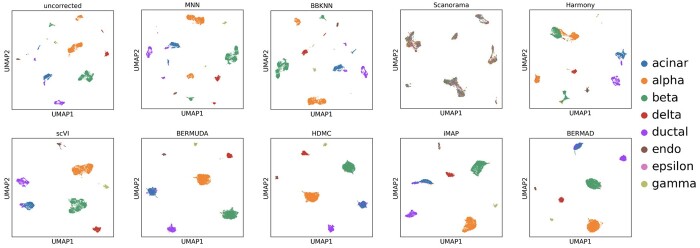
Visualization for batch effect removal on Pancreas_Same dataset.

The visualization results of batch effect correction on Pancreas_Same are shown in Fig. 3. It can be seen that the performance of MNN-based methods (MNN, BBKNN and Scanorama) and dimension reduction-based method (Harmony) are inferior to deep learning-based methods (scVI, BERMUDA, HDMC and BERMAD) when faced with such real datasets. Specifically, MNN and BBKNN were unable to correctly merge cell clusters of the same type from two batches, while Scanorama confused various types of cells together. Therefore, these methods did not work properly on this dataset. Harmony merged some same cell clusters correctly, but there was still significant margin existing between other clusters of the same type. On the contrary, deep learning-based methods are able to perform data integration and batch correction appropriately, and our method achieved the most compact and considerable visualization result among these four methods. At the same time, Harmony, scVI, and BERMUDA all mixed cells of different types to varying degrees, while HDMC and BERMAD avoided such over-correction problem. In addition, the epsilon cells are relatively rare and can be covered by other cells with larger number during plotting process, which may be difficult for visual recognition. This phenomenon is normal and trivial for performance evaluation and visualization analysis.

The calculation results of performance evaluation metrics are presented in Table 2. The quantitative metrics calculation reflects roughly equivalent experimental results to qualitative visualization, and our method BERMAD achieved the best calculation results on all three metrics. The lowest divergence indicates that BERMAD can integrate cell clusters of the same type from different batches most appropriately. The highest silhouette indicates that BERMAD can form the most independent and compact cell clusters, and the highest ARI directly illustrates the clustering accuracy of BERMAD. Note that all other methods have either one or more performance evaluation metrics exhibited worse values, which is consistent with our analysis of their visualization results. On this dataset, we set thr=0.85, α=β=γ=0.2.

**Table 2.** Calculation results of evaluation metrics on Pancreas_Same dataset.

Method	Divergence	Silhouette	ARI
Uncorrected	8.11±0.08	0.37±0.00	0.61±0.00
MNN	7.68±0.07	0.29±0.00	0.50±0.02
BBKNN	7.50±0.00	0.29±0.00	0.55±0.00
Scanorama	2.09±0.00	0.03±0.00	0.00±0.00
Harmony	1.71±0.03	0.21±0.00	0.48±0.00
scVI	1.14±0.02	0.15±0.01	0.68±0.08
BERMUDA	0.48±0.03	0.71±0.01	0.92±0.03
HDMC	0.36±0.03	0.74±0.01	0.93±0.00
iMAP	1.38±0.07	0.49±0.01	0.77±0.00
BERMAD	**0.33 ± 0.03**	**0.80 ± 0.00**	**0.94 ± 0.00**

The optimal results are highlighted in bold font.

### 3.3 PBMC data

To verify the data integration and batch correction capabilities of BERMAD on more complex scRNA-seq datasets, we compared it with comparison methods on PBMC dataset. This is a human peripheral blood monocular cells dataset (Kang *et al.* 2018) where technical variations are confounded with biological signals. 24 679 cells are divided into two groups, one treated with interferon-beta (IFN-β) and the other as a control. Due to different responses of different cells to IFN-β, there is batch effect caused by technical processing between the two groups of data. In the meantime, compared to Pancreas dataset, PBMC dataset has a larger scale, making batch correction tasks on it more challenging.

**Figure 4.**
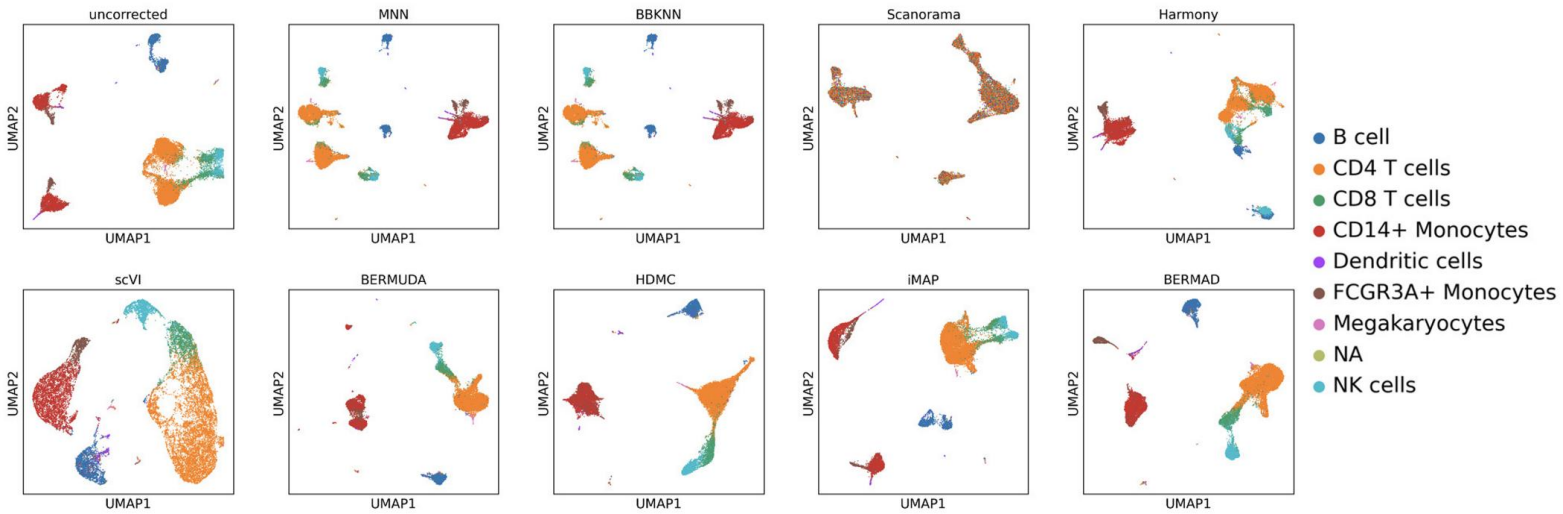
Visualization for batch effect removal on PBMC dataset.

From Fig. 4, BERMAD still produced excellent performance in the face of more complex batch effect. There is significant margin between cell clusters of the same type from the IFN-β treated group and the control group in the uncorrected visualization result, known as batch effect. MNN and BBKNN can only merge CD14+ cells together, while ignoring other types of cells. Scanorama still presented chaotic and disorderly visualization result, mistakenly mixing all types of cells. Although Harmony successfully merged some cell clusters of the same type (CD4 T, CD14+, and FCGR3A+), it caused cell clusters of different types to approach each other (B, CD8 T, and NK), resulting in over-correction problem. iMAP failed to combine all the similar cells between two batches, leaving some cells of the same type (e.g. B and CD14+) far from each other. Compared with the above methods, deep learning-based methods scVI, BERMUDA, HDMC, and BERMAD more accurately integrated cells of the same type while forming more independent cluster structures. However, the cell clusters produced by scVI are relatively loose and far less compact and clear than BERMAD. And there are still varying margins between some cells of the same type (such as CD14+) in the result of BERMUDA, indicating that its data integration and batch correction are not as sufficient as BERMAD. As for HDMC, it mistakenly confused two different types CD14+ and FCGR3A+, which is an over-correction problem. Note that CD4 T, CD8 T, and NK cells are highly similar in biology, so they remain close to each other after correction.

The calculation results of the three performance evaluation metrics on the PBMC dataset are presented in Table 3. It can be seen that all nondeep learning-based methods struggled to achieve better results on all three metrics simultaneously, as their optimization goals usually conflict. On the contrary, deep learning-based methods can improve silhouette and ARI while reducing divergence. Our method BERMAD outperformed all other methods in terms of silhouette and ARI, and its divergence is also lower than most other methods. Although scVI achieved the lowest divergence, its silhouette and ARI were lower than ours, and as mentioned before, its qualitative visualization result was not as good as ours. The above experiment results all demonstrate the effectiveness and superiority of our method on complex datasets such as PBMC. On this dataset, we set thr=0.85, α=β=0.1, γ=0.5.

**Table 3.** Calculation results of evaluation metrics on PBMC dataset.

Method	Divergence	Silhouette	ARI
Uncorrected	5.52±0.03	0.26±0.00	0.50±0.00
MNN	6.44±0.06	0.14±0.00	0.52±0.00
BBKNN	6.40±0.00	0.14±0.00	0.52±0.00
Scanorama	1.01±0.00	0.14±0.00	0.00±0.00
Harmony	4.08±0.01	0.11±0.00	0.37±0.00
scVI	**0.10 ± 0.01**	0.40±0.01	0.59±0.01
BERMUDA	1.53±0.09	0.32±0.01	0.76±0.01
HDMC	0.27±0.04	0.44±0.01	0.68±0.00
iMAP	6.23±0.06	0.19±0.02	0.61±0.09
BERMAD	1.01±0.06	**0.46 ± 0.00**	**0.78 ± 0.00**

The optimal results are highlighted in bold font.

### 3.4 Multiple batch

We also generalized our method to data integration and batch correction for multiple batches, which is more suitable for current application scenario of single-cell analysis. Since BERMAD focuses on cluster pairs between different batches, it can be naturally generalized to multiple batches. Specifically, we find and constrain the similar cluster pairs among all the batches simultaneously. In the meantime, we train an individual autoencoder for each dataset and reconstruct the input by reconstruction loss with the parameters of different autoencoders constrained by transfer loss. We tested BERMAD and all the other comparative methods on Multi-Batch dataset, which consists of three batches named Muraro, Baron and Seger mentioned before. The number of cells in three batches are 1230, 3161, and 1791, respectively.

**Figure 5.**
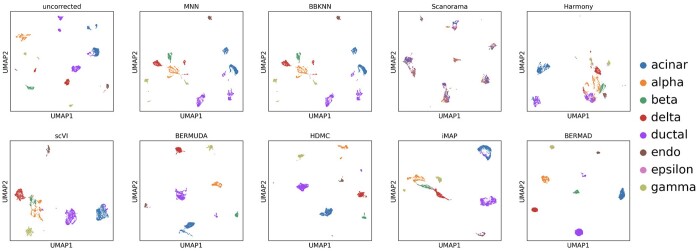
Visualization for batch effect removal on Multi_Batch dataset.

The qualitative visualization results of multi-batch integration are shown in Fig. 5. The quantitative calculation results of evaluation metrics are given in Table 4. From Fig. 5, it can be seen that most compared methods are unable to work well facing more than two batches. They either could not integrate similar clusters between batches correctly, or mistakenly combined dissimilar clusters together. On the contrary, BERMAD managed to remove batch effect between similar clusters and keep dissimilar clusters away from each other, outputting clear and compact clusters. In the meantime, BERMAD achieved best calculation results of all three metrics. Specifically, the lowest divergence means that BERMAD can remove batch effect more sufficiently. The highest silhouette and ARI means that BERMAD can maintain biological specificity better, alleviating over-correction issue. The above experimental results demonstrate our method’s good generalization ability in multi-batch integration scenario.

**Table 4.** Calculation results of evaluation metrics on Multi_Batch dataset.

Method	Divergence	Silhouette	ARI
Uncorrected	7.29±0.04	0.25±0.00	0.38±0.00
MNN	5.75±0.06	0.19±0.00	0.40±0.00
BBKNN	5.73±0.00	0.19±0.00	0.41±0.00
Scanorama	2.34±0.00	0.10±0.00	0.01±0.00
Harmony	3.24±0.03	0.08±0.00	0.27±0.00
scVI	1.13±0.02	0.20±0.02	0.61±0.01
BERMUDA	2.90±0.09	0.70±0.01	0.15±0.00
HDMC	1.11±0.08	0.71±0.03	0.35±0.01
iMAP	1.91±0.04	0.33±0.01	0.47±0.03
BERMAD	**0.34 ± 0.01**	**0.74 ± 0.00**	**0.87 ± 0.00**

The optimal results are highlighted in bold font.

## 4 Ablation studies

In order to verify the efficacy of different components in the framework of our method, we further validate two variants of BERMAD on the ‘Pancreas_Same’ dataset and compare them with the complete framework of BERMAD. Specifically, the two variants are: (i) BERMAD without multi-layer adaptation architecture, which only performs distribution matching on the immediate layer *z* (as shown in Fig. 1), which we refer to as woMultiLayer. (ii) BERMAD without dual-channel framework, where two batches share the same model architecture and parameter settings, which we refer to as woDualChannel. The calculation results of three metrics are shown in Table 5. The visualization results can be found in Supplementary material SVIII.

**Table 5.** Calculation results of evaluation metrics for ablation studies.

Method	Divergence	Silhouette	ARI
woDualChannel	**0.11 ± 0.02**	0.67±0.01	**0.94 ± 0.00**
woMultiLayer	0.50±0.03	0.76±0.01	0.93±0.01
BERMAD	0.33±0.03	**0.80 ± 0.00**	**0.94 ± 0.00**

The optimal results are highlighted in bold font.

From the table, the complete BERMAD framework has the best performance comprehensively. Although woDualChannel achieves lower divergence, its silhouette is much lower than BERMAD, indicating that without dual-channel framework, the method focuses more on distribution matching between batches, but ignores the independence of cluster structure. The dual-channel framework can help to generate clearer and independent cluster structures while ensuring accurate correction, which alleviates over-correction issues. For woMultiLayer, it achieves higher silhouette than woDualChannel, but its divergence is significantly higher, and its ARI is also slightly decreased. It indicates that multi-layer adaptation architecture contributes to more comprehensive and accurate data integration and batch correction. Our method BERMAD combines these two parts to obtain the most outstanding performance.

## 5 Conclusion

In this article, we propose a deep learning-based method BERMAD for batch effect removal of scRNA-seq data. BERMAD is a multi-layer adaptation autoencoder with dual-channel framework. By performing distribution matching between batches in multiple intermediate layers with different dimensions in the autoencoder, BERMAD can consider batch effect in data from different granularities and address the insufficient correction issue in other methods. On the other hand, by inputting data from different batches into different deep autoencoder networks as well as independently training, BERMAD explicitly considers batch specific biological signals in the data, effectively reducing over-correction. Qualitative and quantitative experiments on simulated and real datasets show that BERMAD has better visualization results than the competing methods, and outperforms all other methods in different metrics. In general, our method can accurately and sufficiently integrate cells of the same type between different batches while keeping cells of different types away from each other, proving the effectiveness and superiority of our method. In the future, we will focus on integrative analysis of scRNA-seq data with spatial transcriptomic data (Zhang *et al.* 2022), expanding application scenarios and research depth of our method.

## Supplementary Material

btae127_Supplementary_Data
