# Supplementary material for: BERMAD: batch effect removal for single-cell RNA-seq data using a multi-layer adaptation autoencoder with dual-channel framework
Source: Bioinformatics. 2024 Mar 4;40(3):btae127. doi: 10.1093/bioinformatics/btae127 (PMC10942801; doi:10.1093/bioinformatics/btae127)
Supplement: btae127_Supplementary_Data [file btae127_supplementary_data.zip › Supplementary materials.docx]

# Supplementary materials:

# BERMAD: Batch effect removal for single-cell RNA-seq data using a multi-layer adaptation autoencoder with dual-channel framework

## Method workflow

For understandability and applicability, we describe the workflow of our method BERMAD here. The workflow diagram of BERMAD is shown in Figure S1. In general, the complete workflow can be divided into the following steps:

1. Perform clustering for each dataset individually with Seurat package using Louvain algorithm in R, thus dividing cells into different clusters within each batch.
2. Calculate similarity score between cells from different batches, which will be used to recognize similar cell clusters later.
3. Conduct batch correction and data integration using a deep autoencoder with multi-layer adaptation and dual-channel framework.
4. After training, the output combined file can be used for downstream tasks and analysis. In this paper, that will be clustering with k-means algorithm as well as qualitative and quantitative evaluation.


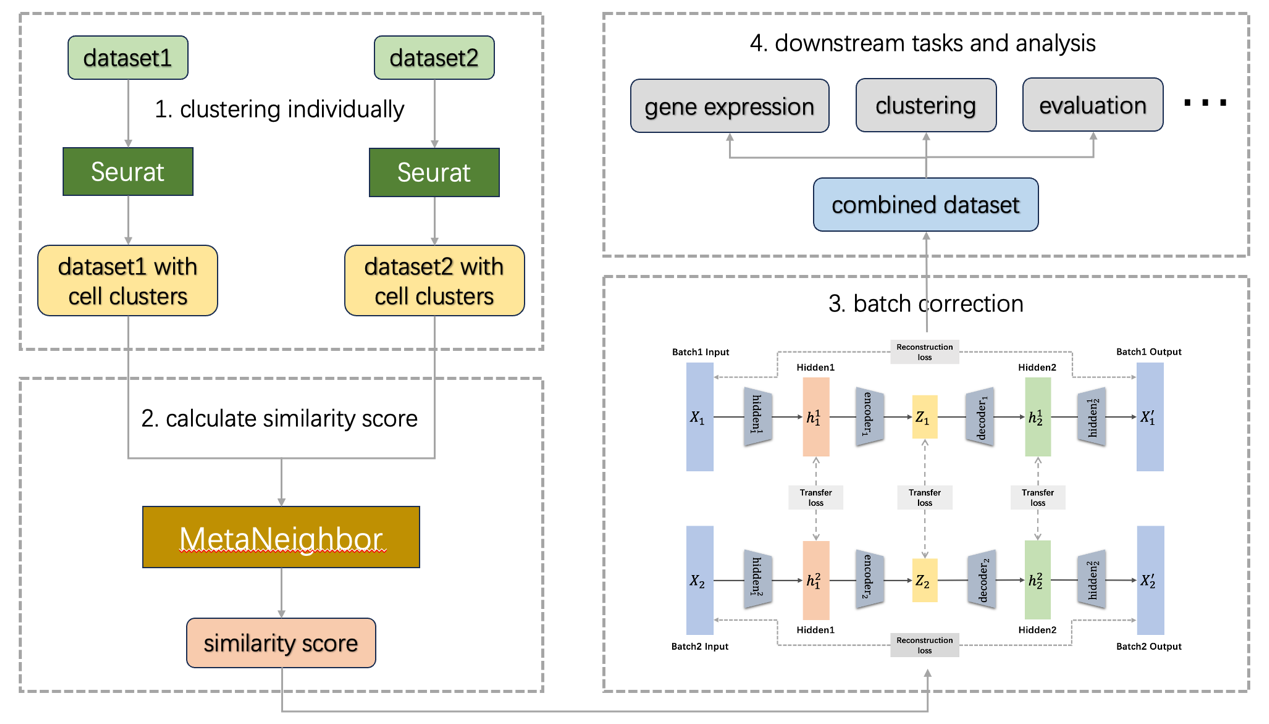


**Fig. S1.** The workflow diagram of our method BERMAD.

## Maximum Mean Discrepancy

The Maximum Mean Discrepancy (MMD) (Gretton et al., 2012) is a non-parametric method to measure the difference between embeddings of the probability distributions in a reproducing kernel Hilbert space (RKHS). It is a kernel two-sample test which rejects or accepts the null hypothesis based on the observed samples (Long et al., 2017). The basic idea behind MMD is that if the generating distributions are identical, all the statistics are the same. It is an effective criterion that compares probability distributions without initially estimating their density functions (Ghifary et al., 2014).

Let $\mathcal{D}_{X^{s}}=\{x_{1}^{s}, \ldots,x_{n_{s}}^{s}\}$ and $\mathcal{D}_{X^{t}}=\{x_{1}^{t}, \ldots,x_{n_{t}}^{t}\}$ be the sets of samples from distributions $P(X^{s})$ and $Q(X^{t})$, respectively. Formally, MMD defines the following difference measure:

$\mathcal{D}_{\mathcal{H}}\left( P,Q \right)\triangleq\sup_{f\mathcal{\in H}} (\mathbb{E}_{X^{s}}\left[ f\left( X^{s} \right) \right]-\mathbb{E}_{X^{t}}\left[ f\left( X^{t} \right) \right])$,

(1)

where $\mathcal{H}$ is a class of functions. In practice, an estimate of the MMD compares the square distance between the empirical kernel mean embeddings as:

${\hat{\mathcal{D}}}_{\mathcal{H}}\left( P,Q \right)=\frac{1}{n_{s}^{2}}\sum_{i=1}^{n_{s}} \sum_{j=1}^{n_{s}} k\left( x_{i}^{s}, x_{j}^{s} \right)+\frac{1}{n_{t}^{2}}\sum_{i=1}^{n_{t}} \sum_{j=1}^{n_{t}} k(x_{i}^{t}, x_{j}^{t})-\frac{2}{n_{s}n_{t}}\sum_{i=1}^{n_{s}} \sum_{j=1}^{n_{t}} k\left( x_{i}^{s}, x_{j}^{t} \right)$,

(2)

Where ${\hat{\mathcal{D}}}_{\mathcal{H}}\left( P,Q \right)$ is an unbiased estimator of $\mathcal{D}_{\mathcal{H}}\left( P,Q \right)$.

In domain adaptation or transfer learning, MMD has been used to reduce the distribution mismatch between the source and target domain (Ghifary et al., 2014). Pan et al. (2010) proposed a PCA-based model called Transfer Component Analysis (TCA), which used MMD to induce a subspace where the data distributions in different domains are closed to each other. Long et al. (2013) presented a Transfer Sparse Coding (TSC) that utilizes MMD in the encoding stage to match the distributions of the sparse codes. In this paper, we utilize MMD to conduct distribution matching between different batches to accomplish batch correction.

## MetaNeighbor algorithm

MetaNeighbor (meta-analysis via neighbor voting) is a simple, supervised framework to assess how well cell-type-specific transcriptional profiles replicate across datasets (Crow et al., 2018). It makes use of the cell-type labels supplied by data providers, and assesses the correspondence of cell types across datasets by taking the following approach:

1. Generate a network about correlations between all pairs of cells.
2. Divide the datasets into training set and test set.
3. Predict the labels of the test set based on the training data.

We use MetaNeighbor to calculate similarity score between cells from different batches, thus integrating cells of the same type together with our method BERMAD.

## Comparative experiments on hyperparameters of hidden layers


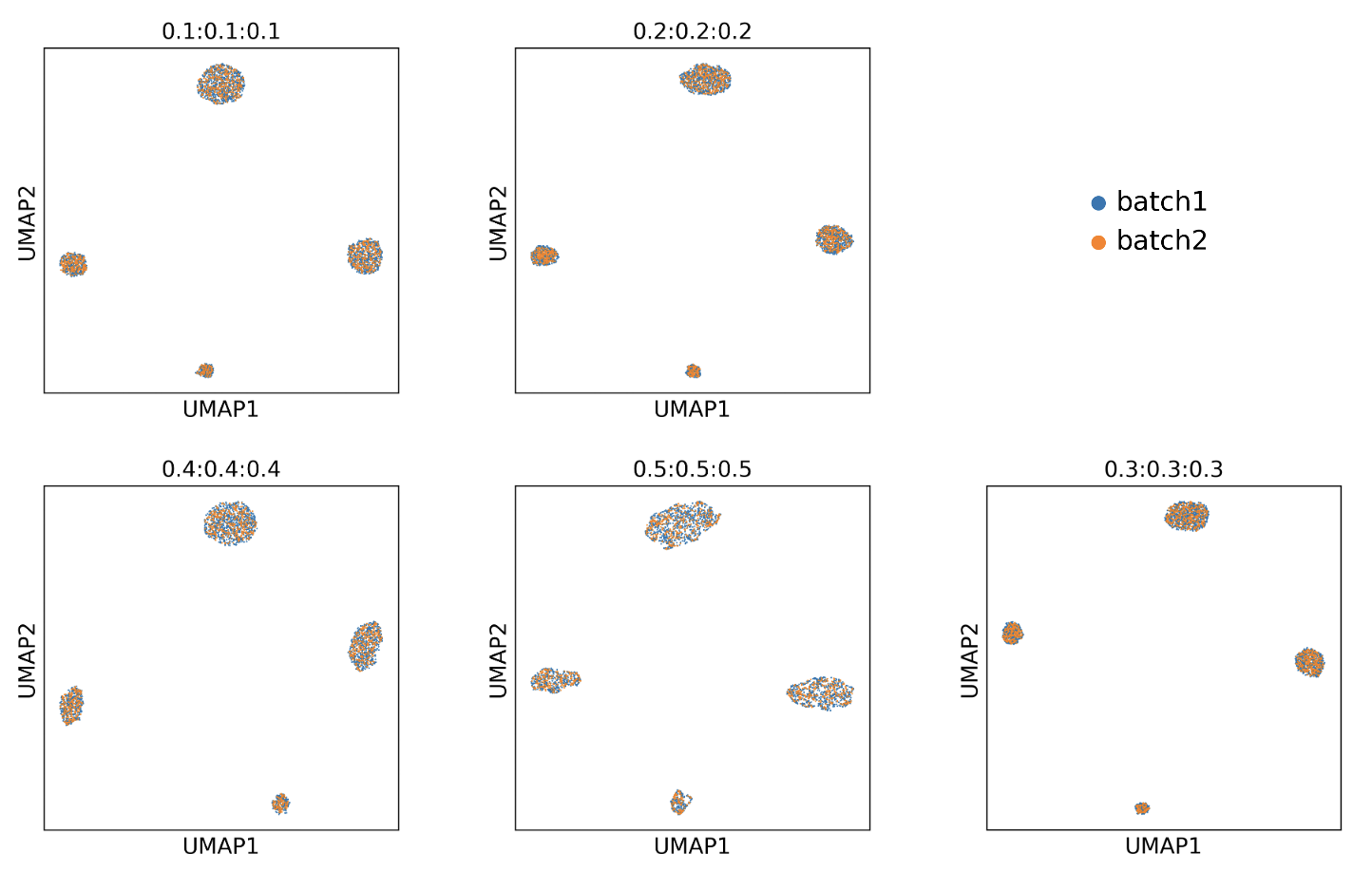


**Fig. S2.** Visualization results of different hyperparameter values on Simulated dataset. Cells are dyed by batch ID.


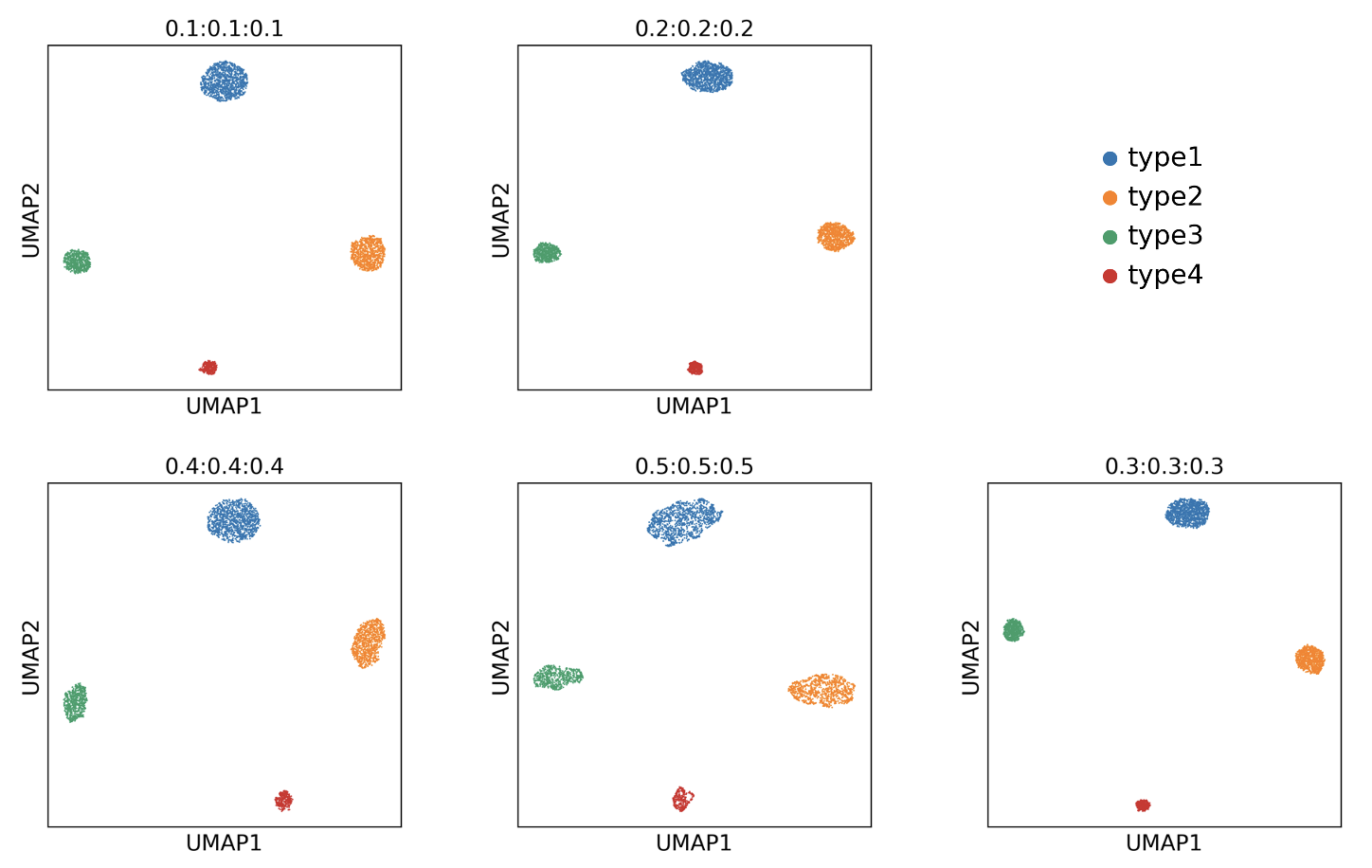


**Fig. S3.** Visualization results of different hyperparameter values on Simulated dataset. Cells are dyed by type.

**Tab. S1.** Metric results of different hyperparameter values on Simulated Dataset.

| $\boldsymbol{\alpha:\beta:\gamma}$ | Divergence | Silhouette | ARI |
| --- | --- | --- | --- |
| uncorrected | 3.96±0.06 | 0.34±0.00 | 1.00±0.00 |
| 0.1:0.1:0.1 | 0.01±0.00 | 0.92±0.00 | 1.00±0.00 |
| 0.2:0.2:0.2 | 0.01±0.00 | 0.94±0.00 | 1.00±0.00 |
| 0.3:0.3:0.3 | 0.01±0.00 | 0.95±0.00 | 1.00±0.00 |
| 0.4:0.4:0.4 | 0.02±0.01 | 0.96±0.01 | 1.00±0.00 |
| 0.5:0.5:0.5 | 0.04±0.00 | 0.99±0.00 | 1.00±0.00 |


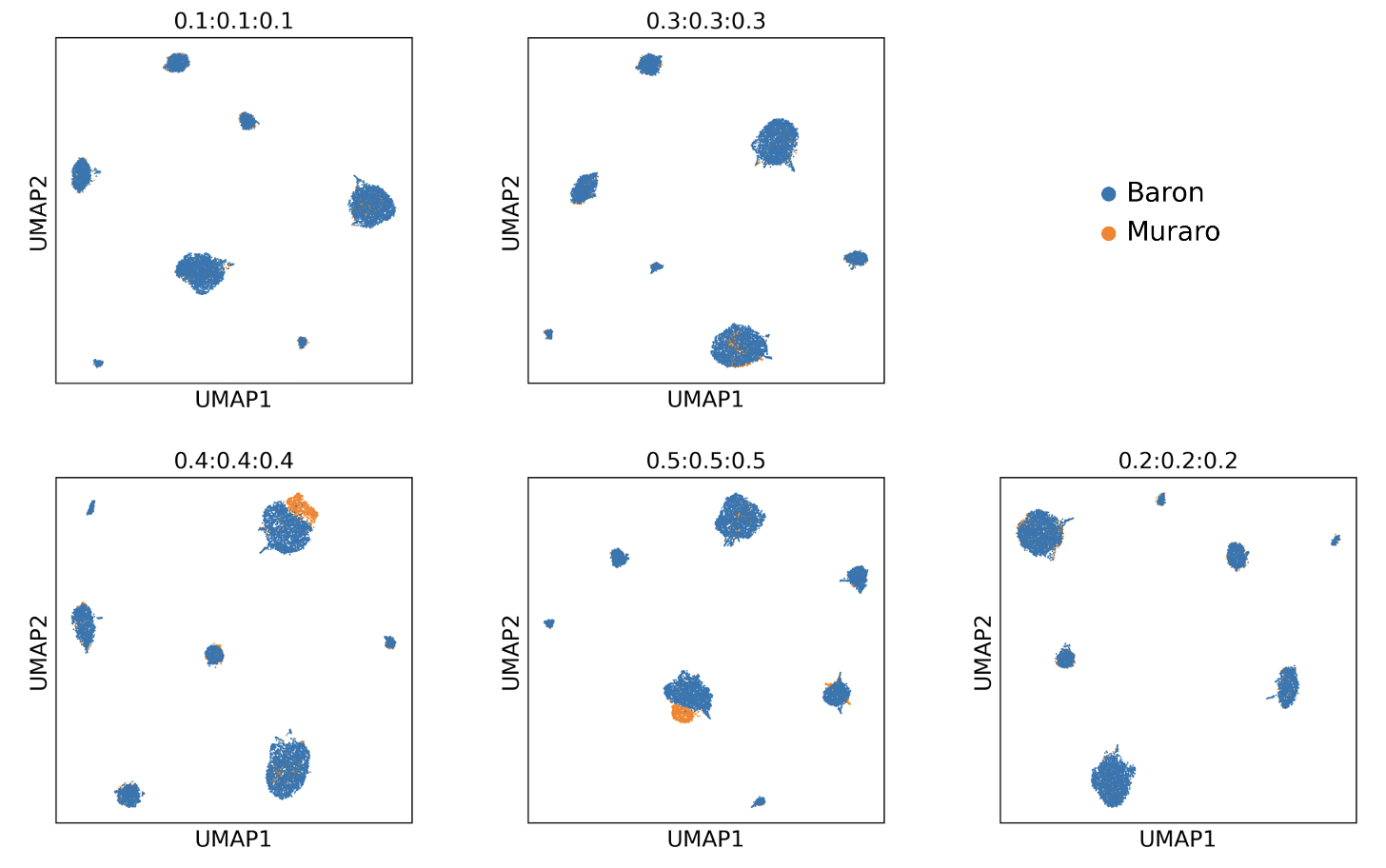


**Fig. S4.** Visualization results of different hyperparameter values on Pancreas dataset. Cells are dyed by batch ID.


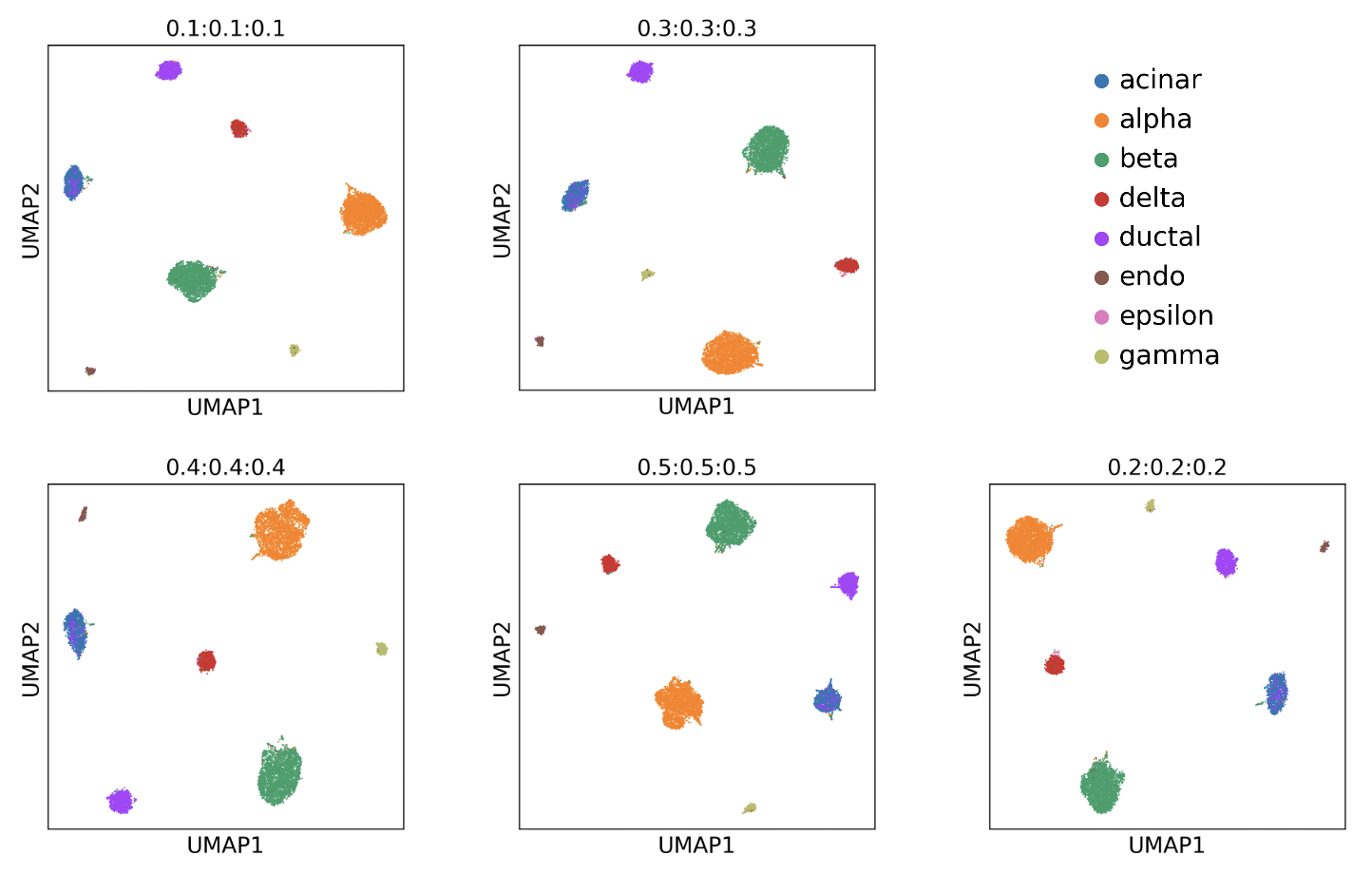


**Fig. S5.** Visualization results of different hyperparameter values on Pancreas dataset. Cells are dyed by type.

**Tab. S2.** Metric results of different hyperparameter values on Pancreas Dataset.

| $\boldsymbol{\alpha:\beta:\gamma}$ | Divergence | Silhouette | ARI |
| --- | --- | --- | --- |
| uncorrected | 8.11±0.08 | 0.37±0.00 | 0.61±0.00 |
| 0.1:0.1:0.1 | 0.13±0.01 | 0.72±0.01 | 0.93±0.00 |
| 0.2:0.2:0.2 | 0.33±0.03 | 0.80±0.00 | 0.94±0.00 |
| 0.3:0.3:0.3 | 0.67±0.02 | 0.81±0.01 | 0.94±0.00 |
| 0.4:0.4:0.4 | 0.77±0.03 | 0.81±0.01 | 0.93±0.00 |
| 0.5:0.5:0.5 | 0.91±0.02 | 0.80±0.04 | 0.92±0.00 |

## Qualitative figures dyed by batch ID


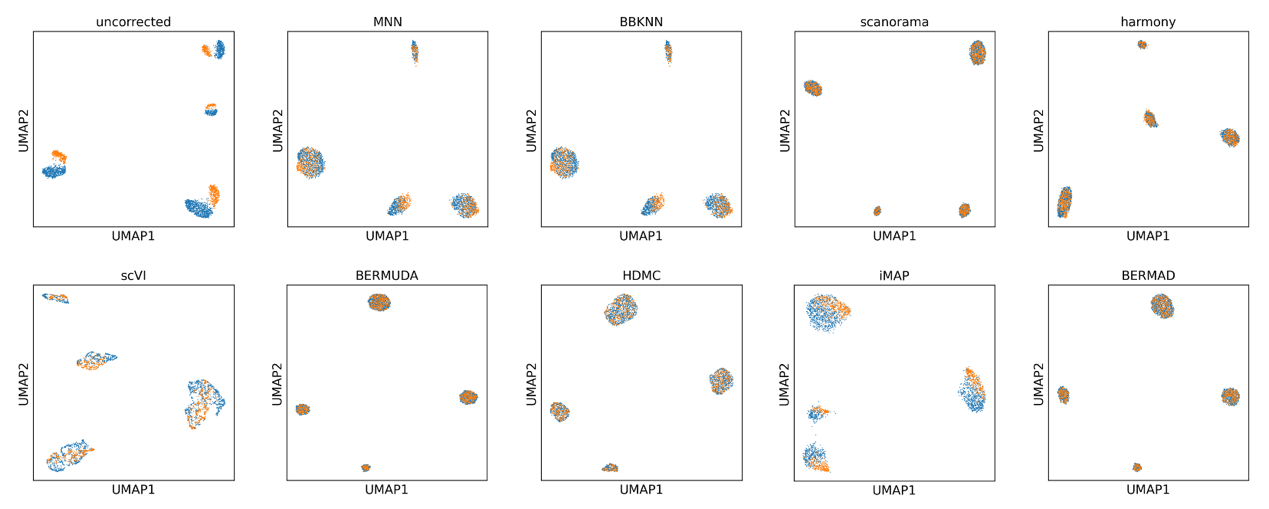


**Fig. S6.** Visualization for batch effect removal on simulated dataset. Cells are dyed by batch ID.


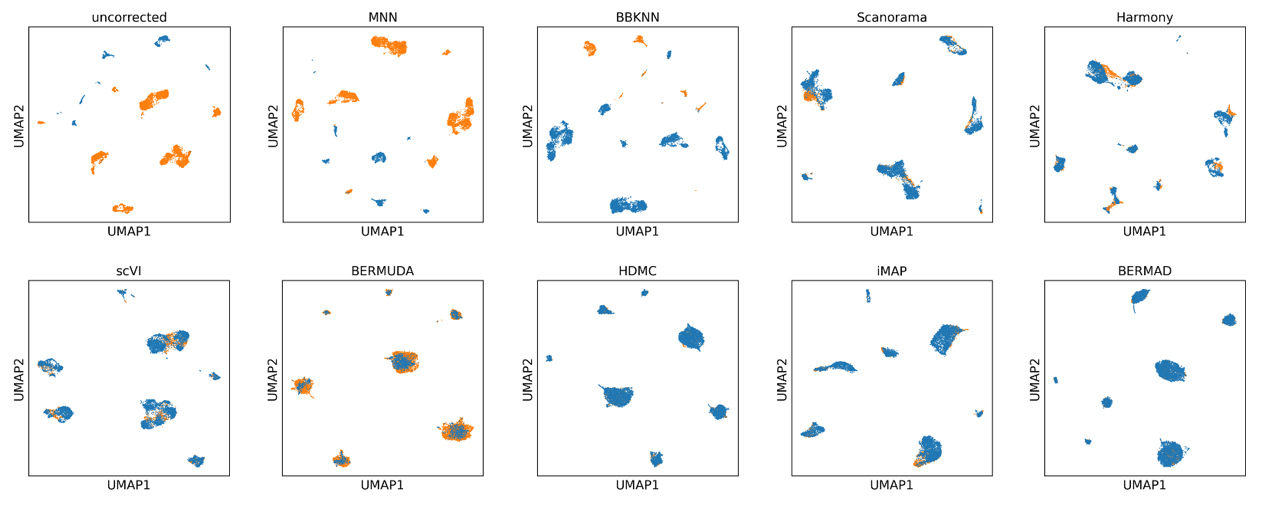


**Fig. S7.** Visualization for batch effect removal on Pancreas_Same dataset. Cells are dyed by batch ID.


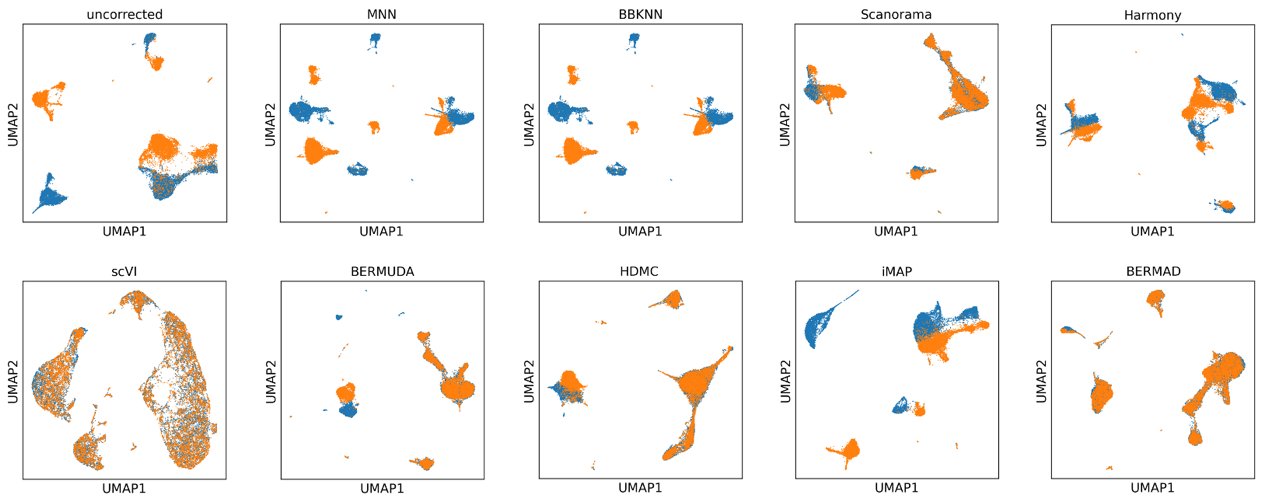


**Fig. S8.** Visualization for batch effect removal on PBMC dataset. Cells are dyed by batch ID.


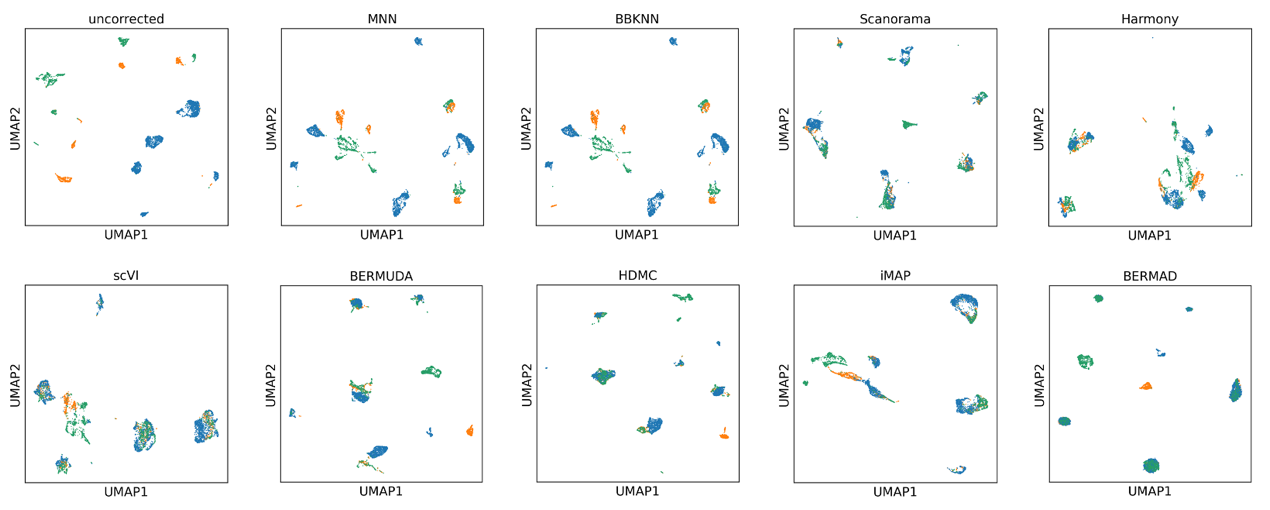


**Fig. S9.** Visualization for batch effect removal on Multi-Batch dataset. Cells are dyed by batch ID.

## Evaluation metrics

We choose three performance evaluation metrics commonly used in this field to evaluate our method from different aspects, and compare BEAMAD with some state-of-the-art methods.

Firstly, because the primary goal of batch correction is to completely integrate cells of the same type between batches, we choose divergence as the first performance evaluation metric, which is used to measure the distribution discrepancy between batches. For $n$ batches $X_{1},X_{2},\ldots,X_{n}$ and their corrected low-dimensional representations $Z_{1},Z_{2},\ldots,Z_{n}$, we refer to Wang *et al*. (2019) to define divergence as follows:

$$divergence=\frac{1}{n_{div}}\sum_{i\neq j} D(s\left( Z_{i},Z_{j} \right),s(Z_{j},Z_{i})),$$

(1)

where $s\left( Z_{i},Z_{j} \right)$ is the cell population in $Z_{i}$ shared by $Z_{j}$, $D$ is the divergence estimation of the two distributions given samples $Z_{i}$ and $Z_{j}$, and $n_{div}$ is the number of batch pairs with shared cell population. Since the goal is to minimize the distribution discrepancy between similar cell clusters, a lower divergence is better.

Secondly, whether dissimilar cell clusters still maintain good separation after integration is also an important factor to consider. We choose silhouette as the measure to evaluate this separation. Following Haghnerdi *et al*. (2018), for cell $c$, let $a(c)$ represent the average distance of $c$ to cells within the same cluster, and $b(c)$ represent the smallest of the average distances of $c$ to all cells in any other cluster. Then the silhouette of cell $c$ is defined as:

$$silhouette\left( c \right)=\frac{b\left( c \right)-a\left( c \right)}{\max\left\{ a\left( c \right),b\left( c \right) \right\}},$$

(2)

We take the average value of all cells’ silhouette as the final evaluation metric. Since the dataset corrected should have a clear cluster structure, which means same cells are close to each other, while different cells are far away from each other, a higher silhouette is preferred.

Finally, to evaluate how much the batch correction can boost the performance of downstream tasks, we use the k-means algorithm to cluster the corrected data, and then calculate the Adjusted Rand Index (ARI) (Hubert *et al*., 1985) commonly used in clustering tasks to evaluate the method:

$$ARI=\frac{\sum_{ij} \binom{n_{ij}}{2}-[\sum_{i} \binom{a_{i}}{2}+\sum_{j} \binom{b_{j}}{2}]\binom{n}{2}}{\left( \frac{1}{2} \right)\left[ \sum_{i} \binom{a_{i}}{2}+\sum_{j} \binom{b_{j}}{2} \right]-[\sum_{i} \binom{a_{i}}{2}+\sum_{j} \binom{b_{j}}{2}]\binom{n}{2}},$$

(3)

where $n_{ij}$ is the number of cells assigned to cluster $i$ and $j$ based on the true labels and the cluster labels, $a_{i}$ is the number of cells assigned to cluster $i$ by the true labels while $b_{j}$ is the number of cells assigned to cluster $j$ by the cluster labels. A higher ARI means that the clustering result is closer to ground truth, which also means better batch correction performance.

## Experimental results on Pancreas_Diff1 and Pancreas_Diff2


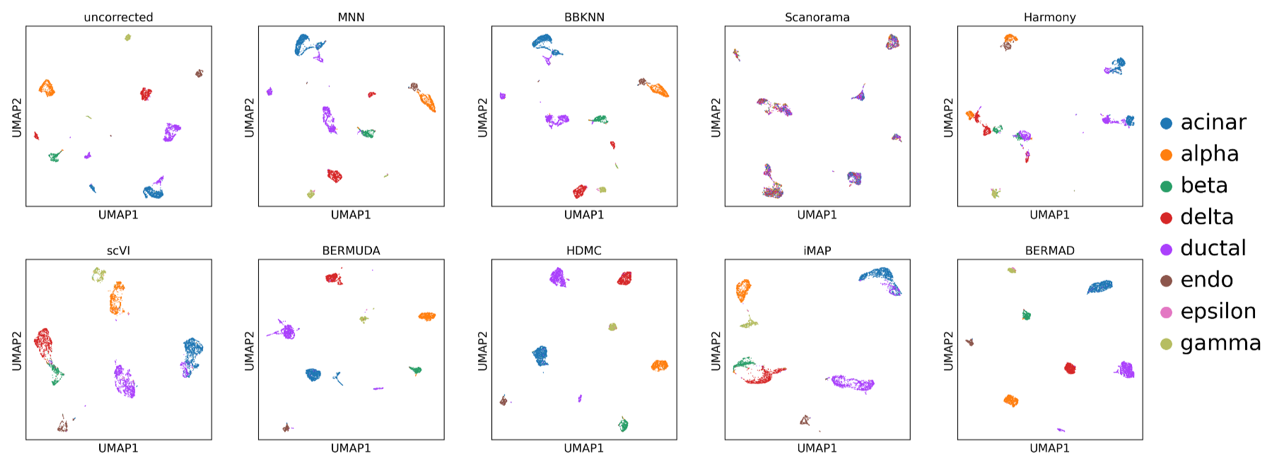


**Fig. S10.** Visualization for batch effect removal on Pancreas_Diff1 dataset. Cells are dyed by type.


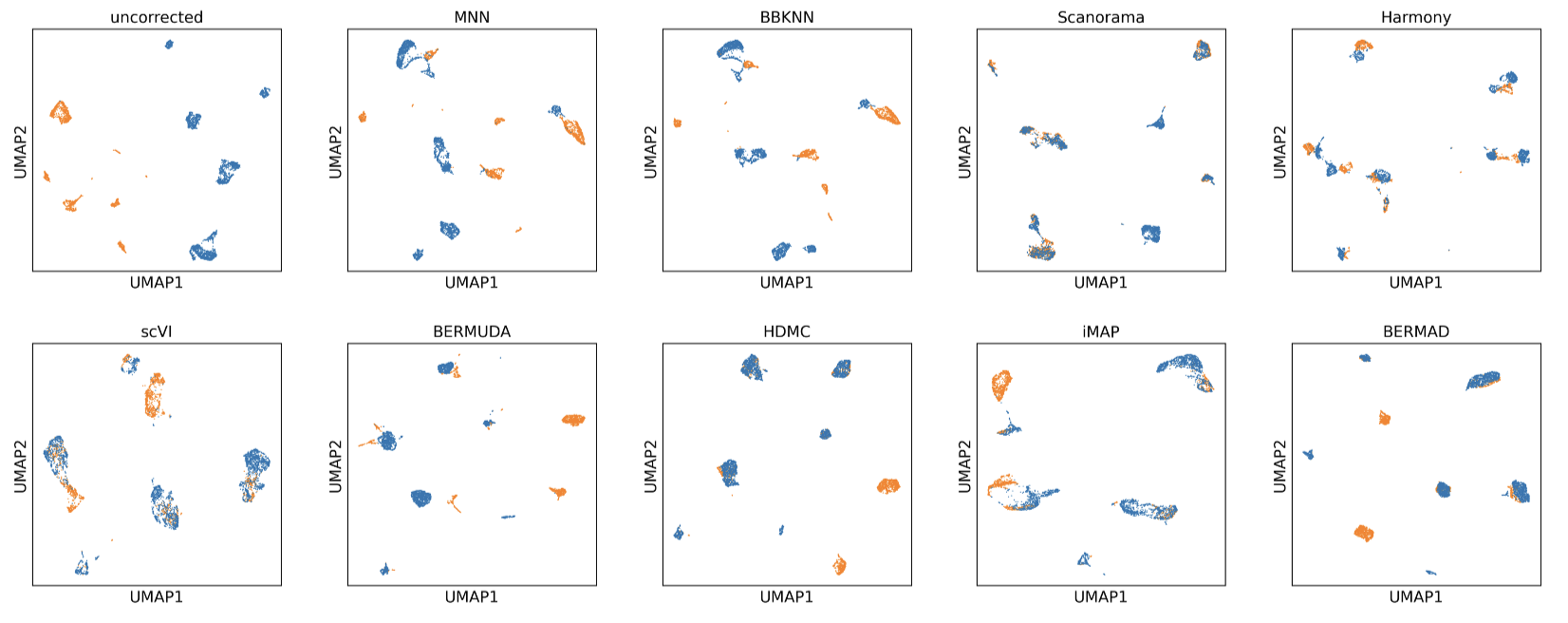


**Fig. S11.** Visualization for batch effect removal on Pancreas_Diff1 dataset. Cells are dyed by batch ID.

**Tab. S3.** Calculation results of evaluation metrics on Pancreas_Diff1 dataset.

| Method | Divergence | Silhouette | ARI |
| --- | --- | --- | --- |
| uncorrected | 8.01±0.08 | 0.42±0.00 | 0.67±0.00 |
| MNN | 5.94±0.00 | 0.22±0.00 | 0.43±0.00 |
| BBKNN | 5.84±0.00 | 0.22±0.00 | 0.43±0.00 |
| Scanorama | 1.51±0.00 | 0.12±0.00 | 0.02±0.00 |
| Harmony | 2.84±0.03 | 0.07±0.00 | 0.29±0.00 |
| scVI | 1.52±0.13 | 0.26±0.01 | 0.74±0.02 |
| BERMUDA  HDMC  iMAP | 2.26±0.08  0.95±0.12  1.71±0.08 | 0.68±0.01  0.66±0.00  0.40±0.02 | 0.48±0.01  0.27±0.00  0.55±0.02 |
| BEAMAD | **0.69±0.08** | **0.74±0.00** | **0.81±0.00** |


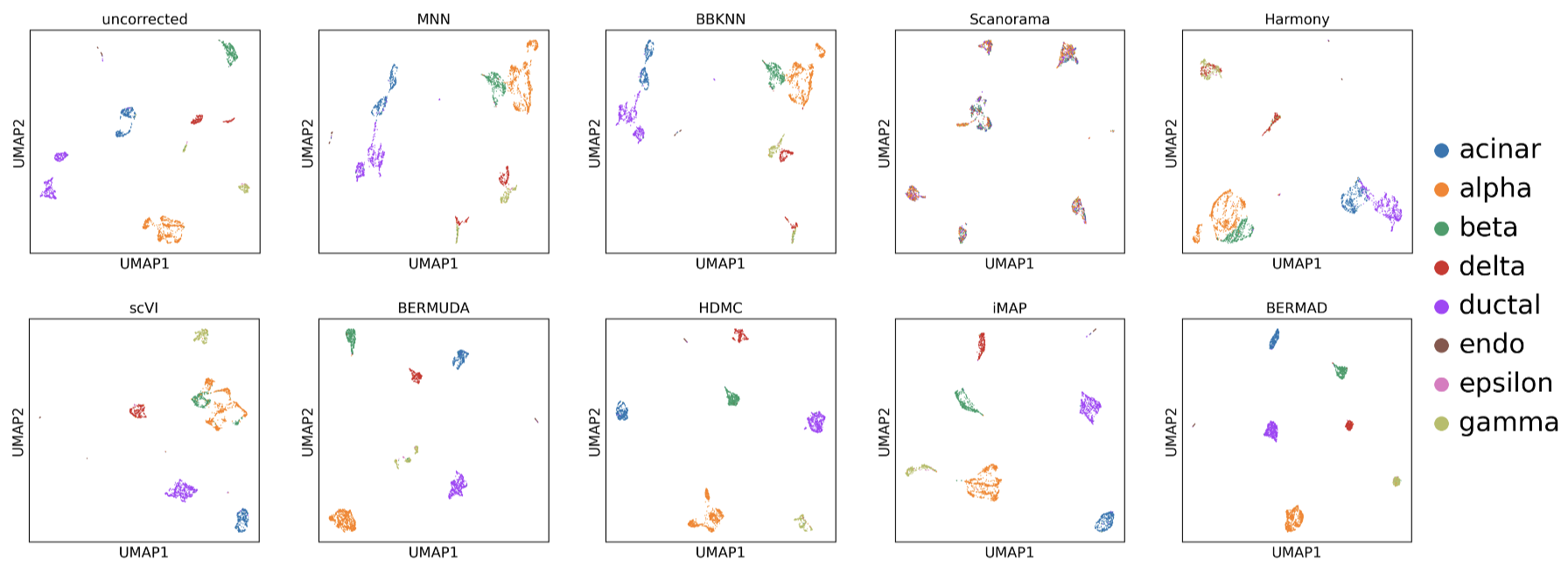


**Fig. S12.** Visualization for batch effect removal on Pancreas_Diff2 dataset. Cells are dyed by type.


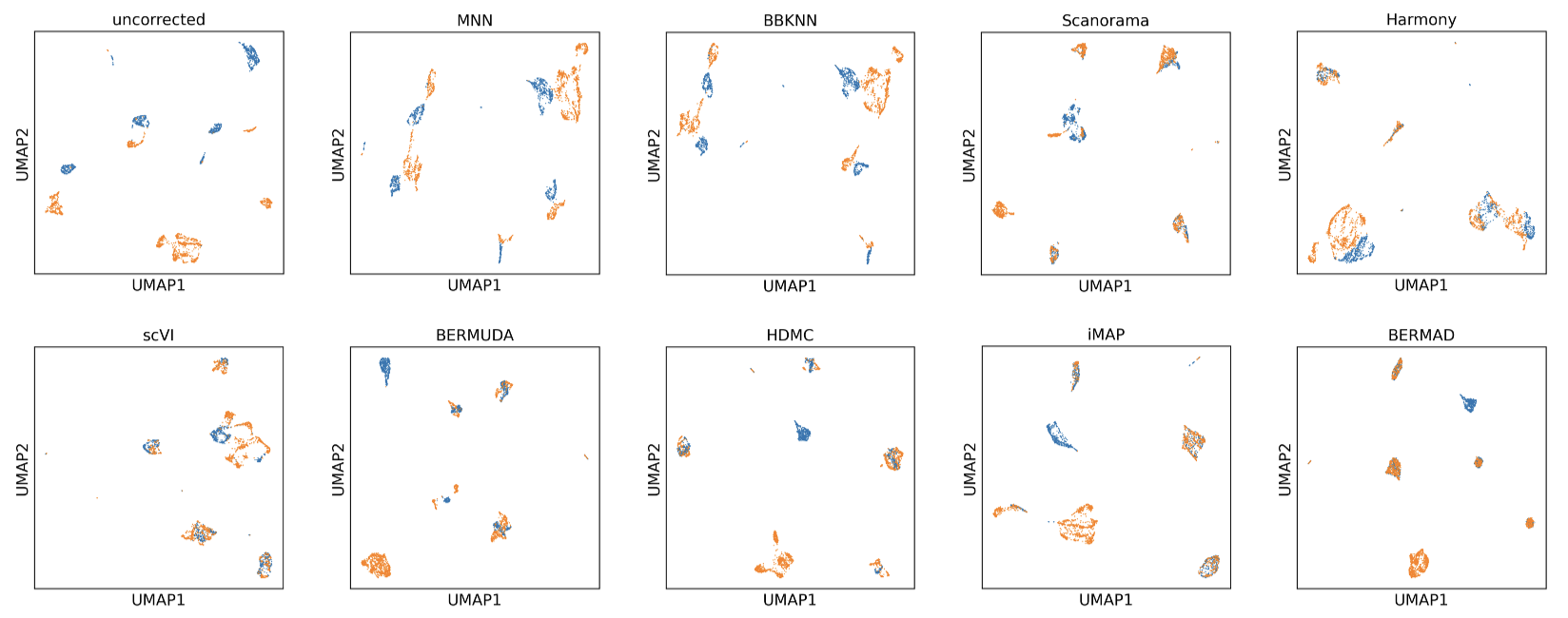


**Fig. S13.** Visualization for batch effect removal on Pancreas_Diff2 dataset. Cells are dyed by batch ID.

**Tab. S4.** Calculation results of evaluation metrics on Pancreas_Diff2 dataset.

| Method | Divergence | Silhouette | ARI |
| --- | --- | --- | --- |
| uncorrected | 5.07±0.12 | 0.36±0.00 | 0.59±0.00 |
| MNN | 3.11±0.00 | 0.12±0.00 | 0.43±0.00 |
| BBKNN | 3.10±0.00 | 0.12±0.00 | 0.41±0.00 |
| Scanorama | 2.23±0.00 | 0.13±0.00 | 0.03±0.00 |
| Harmony | 0.94±0.01 | 0.10±0.00 | 0.41±0.00 |
| scVI | 0.84±0.04 | 0.22±0.01 | 0.68±0.02 |
| BERMUDA  HDMC  iMAP | 2.17±0.07  1.46±0.10  0.80±0.05 | 0.69±0.00  **0.83±0.02**  0.38±0.03 | 0.44±0.09  0.59±0.08  0.46±0.03 |
| BEAMAD | **0.27±0.02** | 0.74±0.00 | **0.81±0.01** |

## Qualitative results of ablation study


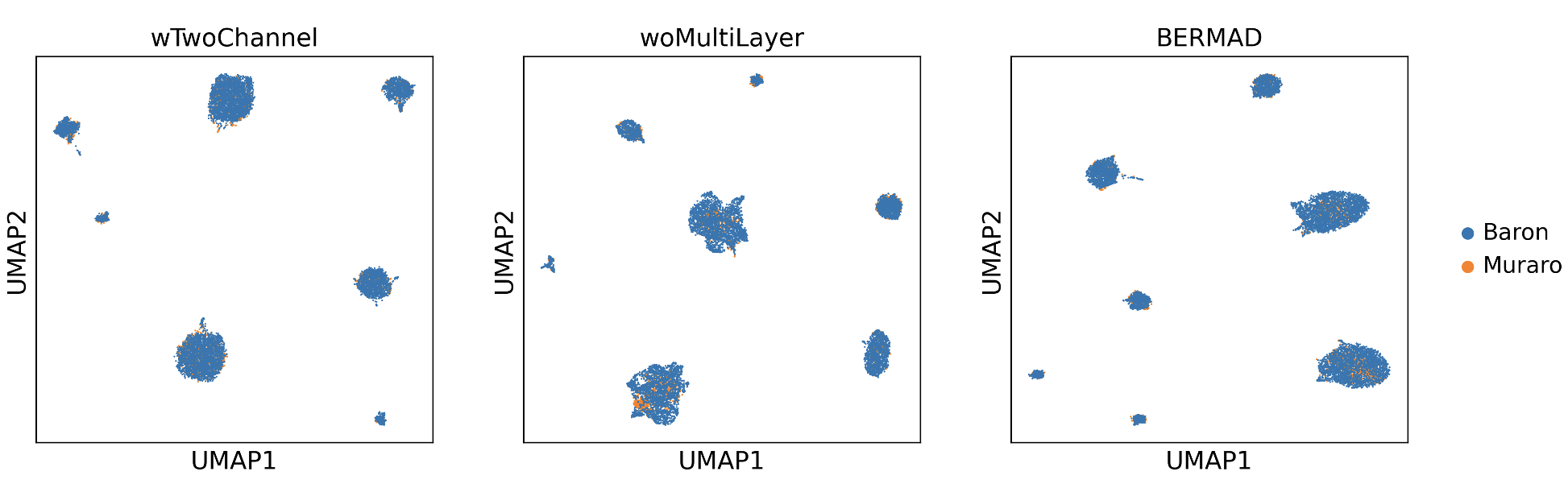


**Fig. S14.** Visualization results of ablation study on Pancreas dataset. Cells are dyed by batch ID.


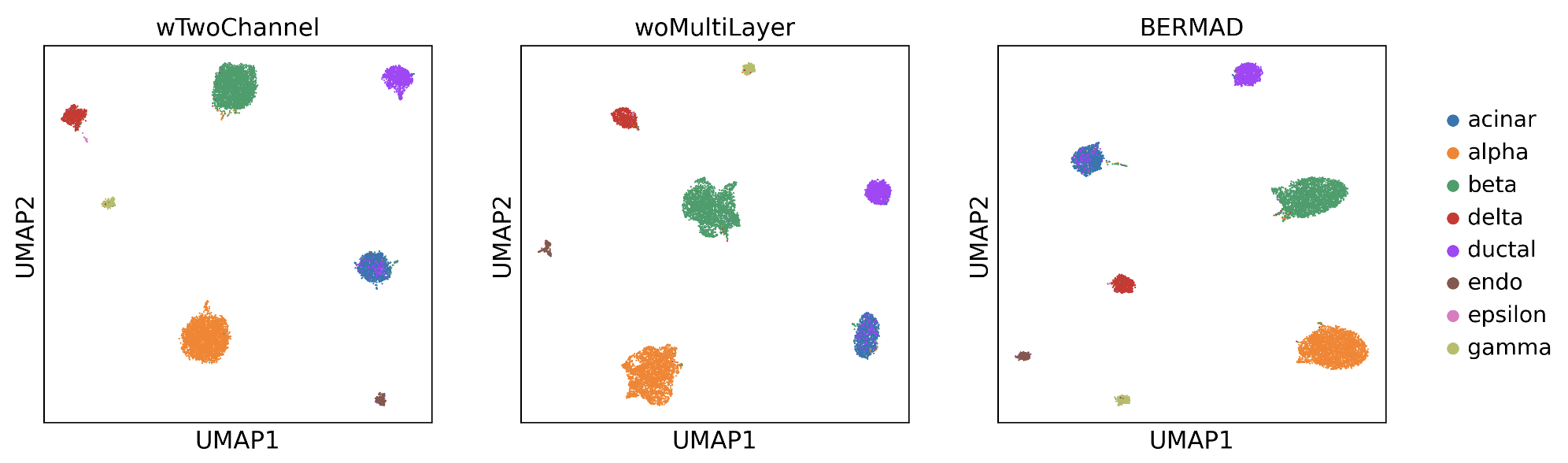


**Fig. S15.** Visualization results of ablation study on Pancreas dataset. Cells are dyed by type.

## References

1. Gretton, A. *et al*. (2012) Optimal kernel choice for large-scale two-sample test. *Advances in neural information processing systems*., **25**, 1205-1213.
2. Long, M *et al*. (2017) Deep transfer learning with joint adaptation networks. *International conference on machine learning*., **70**, 2208-2217.
3. Ghifary, M. *et al.* (2014) Domain Adaptive neural networks for object recognition. *Trends in Artificial Intelligence*., **13**, 898-904.
4. Pan, S.J. *et al*. (2010) Domain Adaptation via transfer Component analysis. *IEEE transactions on neural networks*., **22**, 199-210.
5. Long, M. *et al*. (2013) Transfer sparse coding for robust image representation. *Proceedings of the IEEE conference on computer vision and pattern recognition*., 407-414.
6. Wang, T. *et al*. (2019) BERMUDA: a novel deep transfer learning method for single-cell RNA sequencing batch correction reveals hidden high-resolution cellular subtypes. *Genome Biol*., **20**, 165.
7. Haghnerdi, L. *et al*. (2018) Batch effects in single-cell RNA sequencing data are corrected by matching mutual nearest neighbors. *Nat Biotechnol*., **36**, 421-427.
8. Hubert, L. *et al*. (1985) Comparing partitions. *Journal of classification*., **2**, 193-218.
